# Nesting strategies and disease risk in necrophagous beetles

**DOI:** 10.1002/ece3.3919

**Published:** 2018-02-19

**Authors:** Verônica Saraiva Fialho, Vinícius Barros Rodrigues, Simon Luke Elliot

**Affiliations:** ^1^ Department of Entomology Universidade Federal de Viçosa Viçosa Minas Gerais Brazil

**Keywords:** behavioral immunity, carrion ecology, *Coprophanaeus bellicosus*, dung beetles, Hypocreales, Silphidae

## Abstract

While the effects of carcass decomposition on microorganisms have been demonstrated in recent years, little is known of how this impacts necrophagous insects. A common assumption is that insects that exploit carcasses are exposed to a high density of potentially harmful microorganisms, but no field data have so far validated this. Necrophagous beetles such as the Scarabaeinae have complex nesting behaviors with elaborate parental care. So here, we begin to explore whether this conjunction of life history and nesting behavior represents an adaptive response to the threat posed by microbes in these environments, mainly by entomopathogens. We evaluated the density and distribution of fungi and bacteria from soil near the carcasses, and their ability to infect and kill insects that are in contact with this soil during the decomposition process. Our data showed an increase in the density and activity of opportunistic or facultative pathogens during the apex of decomposition, when there is a predominance of necrophagous insects. Meanwhile, the survivorship of bait insects decreased when in contact with soil from this period of decomposition, indicating a potential risk of infection. However, the density and activity of these microorganisms decreased with distance from the carcass, mainly with depth, which would benefit tunneller beetles in particular. We have thus provided the first field data to show that necrophagous insects are indeed exposed to high densities of potentially harmful microorganisms. Furthermore, we propose that some parental care strategies may have arisen not only as a response to competition, but also as adaptations that reduce the risks of disease. Although we have focused on carrion feeders, we suggest that the same occurs with coprophagous beetles, as both carrion and dung are nutrient‐rich resources.

## 1 INTRODUCTION

The body of a dead animal is a rich, yet ephemeral and unpredictable, source of nutrients for microbial decomposers and detritivorous animals (Barton, Cunningham, Lindenmayer, & Manning, 2013). While microorganisms and insects can be considered the main agents of decomposition, there has been little integration of their effects and even less consideration of how such effects might be affected by interactions between these two groups of organisms. In recent years, microbial decomposition of carcasses has been considered from ecological and evolutionary perspectives (Crippen, Benbow, & Pechal, 2015). To our knowledge, however, no studies have considered the effects of the microbial communities (in particular potential pathogens) on the necrophagous insect community.

**Figure 1:**
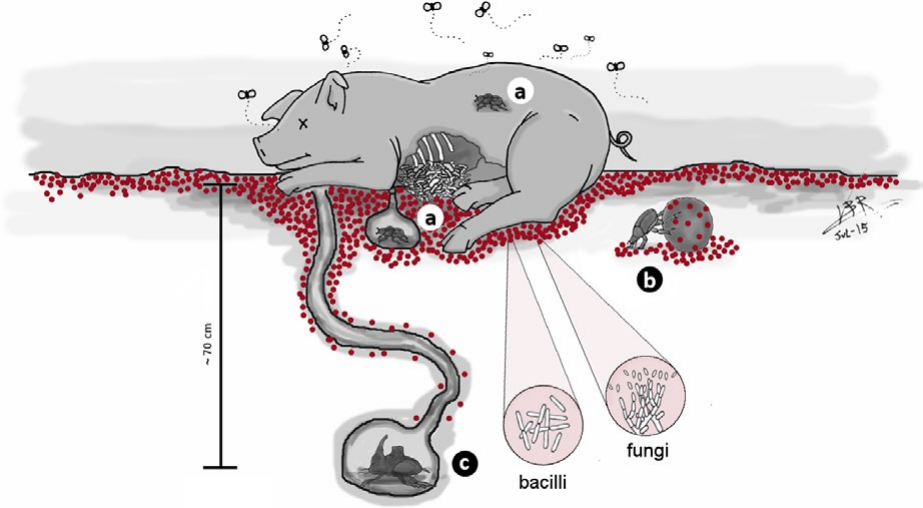
Schematic representation of the microbial distribution around a carcass. Red dots correspond to a general distribution pattern of microbial colony‐forming units in the vicinities of carrion. Note the areas near the surface and deeper in the ground, considering the potential infection risks to which beetles may be exposed when visiting and exploring the carcass and relocating food (i.e., carcass fragments). Note also the relative exposure of the beetles according to their nesting behaviors: (a) dwellers or endocoprids (e.g., *Eurysternus* spp.); (b) rollers or telecoprids (e.g., *Deltochilum (Hybomidium) louzadai*); and (c) tunnellers or paracoprids (e.g., *Coprophanaeus (Megaphanaeus) bellicosus*). Here, the resource is represented by a dead vertebrate, but we can see the same pattern in a dung pile (Halffter & Edmonds, 1982). The vertical bar (ca. 70 cm) represents the mean depth of *C. bellicosus* nests

Carcass decomposition releases large amounts of nutrients and moisture to the ground (Carter, Yellowlees, & Tibbett, 2007; Cobaugh, Schaeffer, & DeBruyn, 2015; Metcalf et al., 2016; Parmenter & MacMahon, 2009; Towne, 2000). This pulse of nutrients changes soil biochemistry and promotes microbial growth, promoting in turn competition and ecological succession (Janzen, 1977; Metcalf et al., 2016). Topsoil microorganisms near to the carcass use these nutrients for growth (Yang, 2004), increasing their activity, and the same might be expected of potentially opportunistic microbes and insect pathogens. Although general effects of carrion decomposition on microorganisms have indeed been shown, most of these studies have had a forensic focus (Burcham et al., 2016; Cobaugh et al., 2015; Finley, Pechal, Benbow, Robertson, & Javan, 2016; Guo et al., 2016; Hopkins, Wiltshire, & Turner, 2000; Howard, Duos, & Watson‐Horzelski, 2010; Olakanye, Thompson, & Komang Ralebitso‐Senior, 2014; Pechal et al., 2013). Considering the facts above, it is clear that insects that exploit carcasses are exposed to an abundant and highly active microbial community (Figure 1). As such, they may be at particular risk of the effects of microbial toxins and deterrent substances (as proposed by Janzen, 1977; and tested e.g., by Burkepile et al., 2006), but also to infection by pathogenic and opportunistic microorganisms. However, to date, this last aspect of carcass ecology has not been studied.

Within necrophagous insect communities, the Scarabaeinae (Coleoptera: Scarabaeidae) are an important and highly diverse group, particularly in tropical forests (Halffter, Favila, & Halffter, 1992; Halffter & Matthews, 1966; Matthews, 1975). These beetles eat and breed in decaying organic matter (dung and/or carrion), contributing greatly to ecosystem functioning, especially with nutrient/energy cycling, and soil bioturbation, which are important for plant growth and water percolation (Nichols et al., 2008). They use the decaying resources as sites to encounter mates, as substrates to make a nest (brood balls) and as food for themselves and their brood (Halffter & Edmonds, 1982). Larvae remain inside the nest for considerable periods, from a few weeks to many months or a year (Anderson & Coe, 1974; Halffter & Edmonds, 1982), which highlights the importance of pathogen‐ and spoiler‐free nesting conditions. A number of laboratory studies with the necrophagous genus *Nicrophorus* (Silphidae) have shown parental care and nest sanitization to be effective strategies in minimizing possible negative effects of microbial competition on larvae (Cotter & Kilner, 2010; Eggert, Reinking, & Müller, 1998; Hall et al., 2011; Rozen, Engelmoer, & Smiseth, 2008). These beetles have complex nesting behavior with elaborate parental care (including regurgitation of predigested food) and removal (burial) of small carcasses as food sources for larvae (Pukowski, 1933; Scott, 1998). This conjunction of life history and nesting behavior has been interpreted as an adaptive response to the threat posed by microbes in these environments (Hall et al., 2011; Rozen et al., 2008). There are, however, no field data (that we have been able to find) to validate this interpretation, despite recent evidence (experimentally in the laboratory) that *N. vespilloides* may manipulate the bacterial community of carcasses (Duarte, Welch, Swannack, Wagner, & Kilner, 2017).

**Figure 2:**
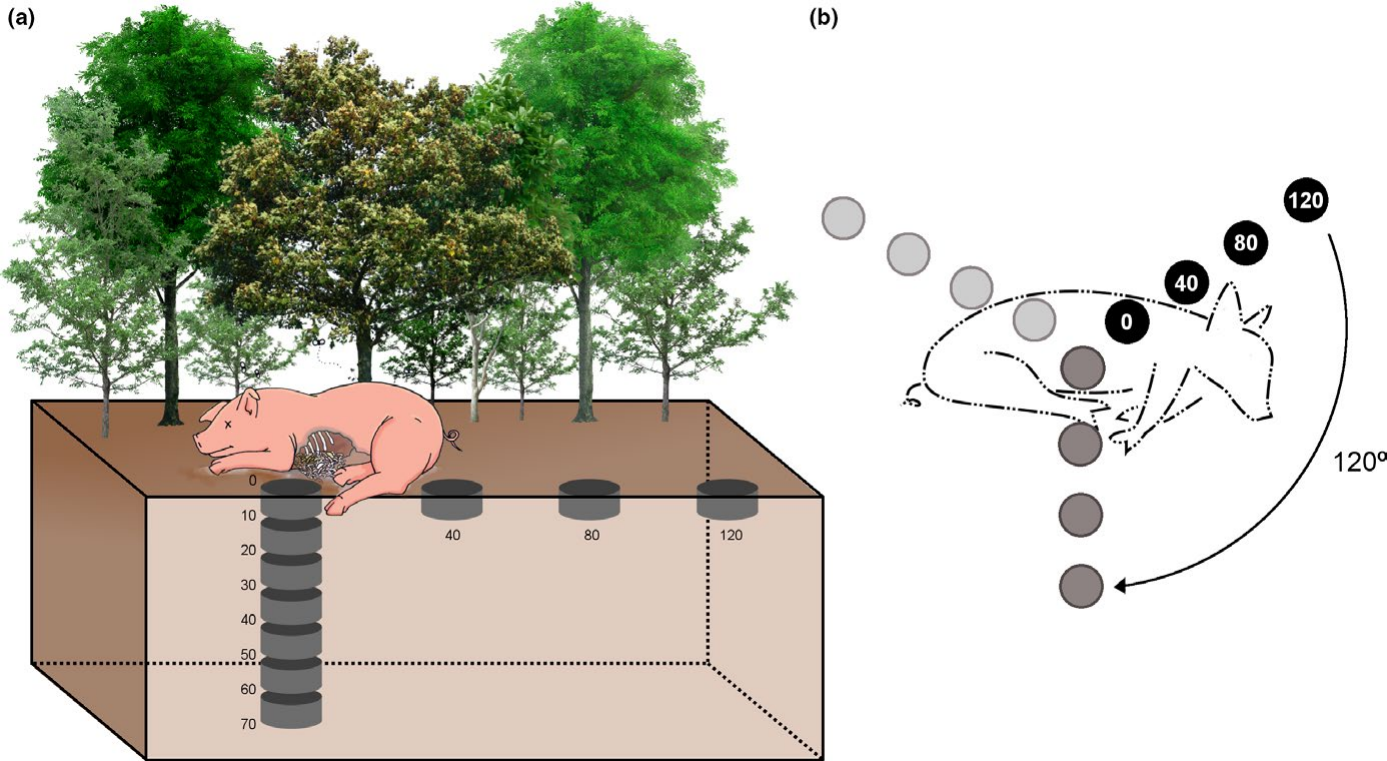
Schematic representation of field sampling to illustrate vertical and horizontal transects with the soil sampling points. Each point corresponds to distances from the pig carcass. These transect directions (a) were defined according to the nesting behaviors of Scarabaeinae: vertical direction for tunnellers (paracoprids) and horizontal direction for rollers (telecoprids), as these are the groups most abundant in Neotropical forests (Halffter et al., 1992). Each sample reached 10 cm depth due to the size of the core soil sampler (a). A schematic view of transects in the above perspective is provided in (b) to show the rotation conducted among the evaluation periods: black circles = “Before” period (before carcass placement); dark gray circles = “During” period (with the carcass in the apex of decomposition); and light gray circles = “After” (after total decomposition of carcass). For the first set of samples (i.e., “Before” period) and for both horizontal and vertical transects within that, a single sample was used per replicate as the first sample (distance 0 cm from pig carcass or origin, so underneath it). For each evaluation period, a new origin was defined as the starting point for both vertical and horizontal transects that went, respectively, down or else horizontally away from the carcass (b); these were taken subsequently on the same date according to distances represented in the scheme. For the subsequent chronological set of samples (evaluation periods), a new origin point was determined to avoid any resampling or effects of experimenter trampling on the soil; the horizontal transects were also moved 120° from the earlier set of horizontal samples (b) to avoid sampling overlap. All vertical transects were performed directly below the carcass (starting from the origin point). The sampling origins were never close to one another than 10 cm. Numbers (a), in centimeters, represent the distances (vertically or horizontally) from the carcass. Tree figures were freely obtained at http://pngimg.com/

Turning to the Scarabaeinae, three main breeding strategies (Figure 1) have been described (Halffter & Edmonds, 1982): (1) endocoprids or dwellers, which nest inside the resource (Bornemissza, 1969) (Figure 1a); (2) telecoprids or rollers that remove a piece of the resource and roll it far away from the origin and hide it superficially in the soil (Figure 1b); and (3) paracoprids or tunnellers that remove a piece of the resource and bury it directly below the resource at different depths (Figure 1c). (Note that the paracoprid strategy (3) has similarities with that of *Nicrophorus* above.) The principal extant hypothesis used to explain this variation in Scarabaeinae nesting strategies is that the relocation of the resource has been selected in response to high levels of intra‐ and interspecific competition to which the beetles would be subject if within the resource (Doube, 1990; Halffter & Matthews, 1966; Janzen, 1977; Scholtz, Davis, & Kryger, 2009). Other factors that may be important are the need for brood protection against predators and parasites (including kleptoparasites, parasitoids, and pathogens) and environmental conditions (Halffter, 1997). Although parental care and relocation of food have high energetic costs, it seems that the benefits of these behaviors outweigh the costs (Favila, 1993; Halffter, Huerta, & Lopez Portillo, 1996). Moreover, some studies had reported that the removal of the parent (and with it parental care) in laboratory conditions causes the growth of fungi on the brood balls (Favila, 1993; Halffter et al., 1996). Similarly, limitation of parental care produces negative effects on *Nicrophorus* offspring (Eggert et al., 1998; Rozen et al., 2008; Schrader, Jarrett, & Kilner, 2015). In the field, however, there have been no reports of infection in Scarabaeinae or their brood balls. We therefore begin to explore, here, the possibility that the elaborate behaviors that are observed in the field result from the selective pressure exerted by microorganisms, in particular, entomopathogens.

We set out to investigate whether the presence of a carcass and its decomposition affect the abundance and activity of putative pathogenic microbes present in the soil. The main questions were as follows: (1) Does carcass decomposition affect the bacterial and fungal communities that might be potentially harmful to insects? (2) How does decomposition affect abundance and activity of these microbes? (3) Do the effects of decomposition decrease with distance from carcass?—vertically and horizontally, considered in accordance with the tunneller and roller lifestyles; (4) do these effects affect in turn the survivorship of coleopteran insects exposed to these local environments? Our biological hypothesis is that the nutrients released by carcass decomposition affect the soil microbial community of putative entomopathogens, increasing their abundance and activity, so constituting an increased risk of disease. We focused on necrophagous and copro‐necrophagous Scarabaeinae to understand the ecology of entomopathogens under carrion decomposition, but discuss implications of our results also for coprophagous beetles, due to similarities in the nutritional richness of both resources (carcass and dung). We focused on two of the behaviors described above—(2) telecoprids (rollers) and (3) paracoprids (tunnellers), as these are more abundant in tropical forests (Halffter et al., 1992). Nevertheless, implications for (1) endocoprids (dwellers) are discussed below, along with suggestions for new investigative avenues.

## 2 MATERIALS AND METHODS

### 2.1 Field site

The experiment was conducted in the research station *Estação de Pesquisa, Treinamento e Educação Ambiental Mata do Paraíso* (20°48′18″S, 42°51′20″W; Appendix S1), run by the Universidade Federal de Viçosa, Viçosa, State of Minas Gerais, southeastern Brazil. This research station (ca. 200 ha) consists of a fragment of Atlantic Forest (435 ha total) of high biological importance, due to rare and threatened species of endemic fauna and flora (Drummond, Martins, Machado, Sebaio, & Antonini, 2005). The mean altitude is 650 m and the climatic classification according to Köppen‐Geiger is Cwa, which corresponds to humid subtropical climates with dry winters (between April and September) and wet summers (between October and March, with 90% of annual rainfall). The soil is classified as red‐yellow latosol, and the clay content is about 35% (ca. 10 cm) increasing with depth to about 70% (ca. 100 cm) (Souza, 2011). A previous report showed this fragment of Atlantic Forest to have a high diversity of Scarabaeinae (Louzada & Lopes, 1997). Some of these beetle species are geographically limited to few localities, but are present in this fragment, such as the necrophagous tunnellers *Dichotomius (Luederwaldtinia) louzadai* Nunes, Carvalho, & Vaz‐de‐Mello, 2016 (Nunes et al., 2016), and *Coprophanaeus (Megaphanaeus) bellicosus* (Olivier 1789) (Edmonds & Zidek, 2010; Maldaner et al., 2017); as well as the rollers *Deltochilum (Hybomidium) louzadai* González‐Alvarado & Vaz‐de‐Mello, 2014 (González‐Alvarado & Vaz‐de‐Mello, 2014) and *De. (Parahyboma) furcatum* (Castelnau 1840) (Almeida, Corrêa, & Grossi, 2015), the latter of which was reported as the most abundant of these necrophagous Scarabaeinae in the first study of this community (Louzada & Lopes, 1997) and remains so to this day (VSF *pers. obs*.).

### 2.2 Experimental approach and design

We set up an experiment to investigate the effects of the decomposition of a nutrient‐rich resource (pig cadavers) on potential pathogens of insects present in the forest soil. To do so, we measured (1) the natural abundances of cultivable putative entomopathogens and opportunistic microbes through time and with distance from the cadaver and (2) the mortality of bait larvae placed in contact with soil samples taken from near the carcasses. The latter measure estimates the real probabilities of infection by microbes. This twin focus, on both abundance and entomopathogenic activity of microorganisms, is intended to illuminate selection pressures on the different nesting behaviors among Scarabaeinae.

Each replicate consisted of a pig carcass (*Sus scrofa* Linnaeus; ± 15 kg), with approximately 150 m distance between each, placed directly on the soil surface in a flat area; each carcass was protected with a metallic mesh cage (19 × 19 mm mesh aperture) to prevent scavenging by vertebrates. All pigs used in this study had died of natural causes and were donated to us by *Granja de Melhoramento de Suínos* of the Department of Animal Husbandry, Universidade Federal de Viçosa.

A pilot experiment (a single pig carcass) was conducted (from late April to the first of June, 2015; dry season, Appendix S2) to test and adjust the methodology, to determine the best periods to monitor the decomposition process, and to determine the number and distances of samples (Appendix S3). These choices were based on characteristics of the forest environment and of the local fauna (e.g., care taken to avoid vertebrate scavengers; mean distances that beetles buried or roll food).

The full experiment was conducted within the wet season (see above), from late January to late February 2016. As tropical forest Scarabaeinae are most abundant and are actively reproducing during this season (Halffter & Edmonds, 1982; Halffter & Matthews, 1966), we consider this period to be of most biological relevance. There were three replicates (with carcasses) and a single control (without carcass). Three distinct periods of evaluation were set up to examine the effects of carrion decomposition on microorganisms: (1) “Before,” by which we mean the evaluation period immediately prior to placement of the “fresh” pig carcass in the field (thus, 0 days after the start of decomposition), that was also considered as a control; (2) “During,” which corresponds to the period of peak decomposition or the “active decay” phase (5–8 days after the start of decomposition); and (3) “After,” which was performed after total decomposition of carcasses or the “skeletonization” phase (30 days after the start of decomposition). [Note that “fresh,” “active decay,” and “skeletonization” are recognized phases within the relevant literature (Byrd & Castner, 2010; Catts & Goff, 1992; Dix & Graham, 2000; Gennard, 2012)]. Within these periods of evaluation, beetles are especially abundant in the “active decay” phase (VSF, unpublished data). Sampling dates and meteorological conditions (precipitation, temperature, and humidity) during the sampling period are shown in Table S1 (Appendix S2).

### 2.3 Soil sampling

Soil samples were taken from predefined positions respective to the carcasses as explained in Figure 2. This sampling was conducted to assess the potential risk of infection that microorganisms might represent to necrophagous beetles that visit the carcass and relocate parts of the carcass as a component of their nesting behaviors. We collected soil in each evaluation period using a core soil sampler (10 cm depth and 10 cm diameter), which was thoroughly cleaned with 70% ethanol between each sampling event to limit cross‐contamination. Samples were placed individually in sterile plastic bags and sealed for transportation. Two transects per replicate were defined: horizontally, to represent the trajectory of roller beetles removing material from the carcass (see above), with four points at 0, 40, 80, and 120 cm from the carcass (measured from the carcass to the center of soil core sampler); and vertically, to represent the trajectory of tunneller beetles from the carcass (see above), with seven distance intervals beneath the carcass, from 0–10 cm (the height of the core sampler) to 70 cm below the carcass (Figure 2a). Due to the size of the core soil sampler, all samples reached a depth of 10 cm. In both cases, maximum distances were based on previous field observations of the beetles' nesting behaviors and nest distances from cadavers. Full details are given in Figure 2.

In the laboratory, leaves and roots were removed and each soil sample was homogenized by manual shaking for 1 min. Subsamples (5 g each) were placed in sterile conical screwcap centrifuge tubes for preparation of soil suspensions to be used for direct microbial isolation from subsamples (see below), while another, substantially larger, portion was used to fill 200‐ml polypropylene containers (also sterile) with perforated ventilation lids for the insect bait assays described below.

### 2.4 Direct microbial isolation from soil

Direct isolation of microbes from soil involved suspending the 5 g soil samples (see above) in 45 ml of sterile aqueous solution (i.e., a 10‐fold dilution) with 0.01% polysorbate 80, vortexing these suspensions for 15 s, and plating on to solid culture media (pour plate method) for counts of colony‐forming units (CFU).

We evaluated the natural occurrence of opportunistic entomopathogenic Hypocreales (Ascomycota) fungi that might be cultivable on artificial medium and have little host specificity (Inglis, Enkerli, & Goettel, 2012). Many hypocrealean fungi are generalist pathogens of insects and have high diversity in tropical forests (Evans, Elliot, & Hughes, 2011; Inglis et al., 2012), where Scarabaeinae are also diverse (Halffter & Edmonds, 1982; Halffter & Matthews, 1966; Halffter et al., 1992). So, we used a selective medium (10 g peptone, 20 g dextrose, 15 g agar, and 1 L distilled water) (Keller, Kessler, & Schweizer, 2003) to isolate CFUs of entomopathogenic hypocrealean fungi from soil samples. After sterilizing the medium, we added a blend of antibiotics (0.05 g/L of cycloheximide, tetracycline, and 0.6 g/L streptomycin) and CTAB (0.2 g/L) as a fungicide (Kepler, Ugine, Maul, Cavigelli, & Rehner, 2015; Posadas, Comerio, Mini, Nussenbaum, & Lecuona, 2012) to promote growth of hypocrealean fungi while inhibiting growth of saprophytic fungi and bacteria. Each plate, with two replicates, was inoculated with 100 μl (spread with a sterile Drigalski spatula) of the stock soil suspension (10^−1^) and stored in the dark at 26°C for 10 days until CFU counts could be made (Kepler et al., 2015).

We performed two different bacterial isolations to obtain general information on the effect of the decomposition process on the soil bacterial community near the carcass. Thus, we isolated cultivable nonfastidious bacteria, which can grow in nutrient agar medium without additional nutritional supplements, and endospore‐forming bacteria from the class Bacilli, which comprises many entomopathogenic *Bacillus* spp. (Fisher & Garczynski, 2012; Jurat‐Fuentes & Jackson, 2012; Tanada & Kaya, 1993). A previous assay using aliquots of the stock soil suspension (10‐fold diluted) was conducted to establish the best dilution for each bacterial group, so as to obtain plates on which CFUs could easily be identified and remain discrete. For the first bacterial isolation, that is, nonfastidious bacteria, we took 1 ml aliquots from the soil suspensions (10^−1^) and diluted these three times in sterile distilled water (10^−4^ final dilution), and then plated 100 μl of the soil dilution in nutrient agar medium plates. For the bacilli, we performed a heat treatment before plating (Fisher & Garczynski, 2012): 1 ml of the soil suspension (10^−1^) was heated in a dry bath incubator at 80°C for 10 min and then chilled on a microtube cooler for ca. 2 min. This methodology kills non‐spore‐forming bacteria and vegetative cells of bacilli, leaving only endospores of bacilli (the main infective or virulent propagules), as these are heat‐resistant and survive the heat treatment, then to germinate. Thus, the samples were diluted twice in sterile distilled water (10^−3^ final dilution), and 100 μl was plated on nutrient agar medium plates (in duplicate). All plates were incubated for 24 h at 26°C in aerobic conditions for subsequent counting of CFUs.

The concentration of CFUs per gram of soil was given by multiplying the number of counted CFUs by 100, as 100 μl of the soil suspension contains 0.01 g soil.

### 2.5 Indirect microbial isolation from soil: insect baiting

We used healthy mealworm larvae (*Tenebrio molitor* Linnaeus 1758, Coleoptera: Tenebrionidae) as bait insects (Keyser, De Fine Licht, Steinwender, & Meyling, 2015; Zimmermann, 1986) to verify the presence of entomopathogens and longevity of bait insects or killing power of microorganisms in the soil samples (Moreira, 2012). As *T. molitor* is not a soil‐dwelling organism, it may be expected to have reduced defenses against soil‐inhabiting pathogens, a desirable trait to test insect susceptibility (potential risk) to infection in this environment. Four larvae (11–15 mm) were placed on the soil of each container; after this, the containers were placed upside down to stimulate larval movement. The containers were incubated at 25 ± 1°C until all larvae had died. During the first 10 days, all containers were shaken daily (repeated inversions) to make the larvae move through the soil (Inglis et al., 2012). When the soil sample was dry, we moistened it by spraying sterile water to maintain humidity. Every two days, the insect baits were reviewed to separate dead insects and avoid secondary infections. All dead insects were recorded for survival analyses until the last one died. The dead mealworms were individually surface‐sterilized for isolation of entomopathogenic fungi within the cadavers and stored in a moisture chamber (microtube half‐filled with moist cotton) in the dark at 26°C for 10 days. After this time, when fungi emerged from insects' bodies, we isolated these with sterile needles on to plates with potato dextrose agar medium (Sigma‐Aldrich, Inc.) with 0.05 g chloramphenicol (diluted in 10 ml of 95% ethanol) per liter of medium to avoid bacterial contamination and incubated these plates in the dark at 26°C for 14 days. The cultures were identified morphologically to genus using taxonomic keys (Humber, 2012a). Isolates were stored at 10°C and preserved in silica gel (Humber, 2012b). The insects from which no fungi emerged (usually with bacteria or nematodes present) were recorded as “other”.

### 2.6 Statistical analyses

All statistical analyses were performed in *R* statistical software (R Core Team 2016). The periods of evaluation (i.e., “Before,” “During,” and “After”) were used as an explanatory variable to construct maximal models in all analyses. We first aimed to determine the effects of the categorical explanatory variable “evaluation periods” (x‐var1) and the continuous explanatory variable “distance from carcass” (x‐var2), that is, depths or horizontal distances, on the continuous response variable “CFU” (y‐var). We analyzed these effects on the three different types of CFU from soil samples: hypocrealean fungi, cultivable nonfastidious bacteria, and bacilli. Thus, six statistical models were constructed independently for the three microbial groups and for depths or horizontal distances from the cadaver. CFU data (densities) were expressed as total numbers of fungal or bacterial CFU per gram of soil. To analyze the CFU counts, including control samples, we used generalized linear models (GLM) with Poisson errors, correcting for overdispersion when necessary (Crawley, 2007). Model simplifications were performed by contrast analysis using chi‐squared tests (for testing hypotheses with count data; Crawley, 2007), amalgamating the terms up to changes in deviance at *p *<* *.05, to obtain the minimal adequate model (Crawley, 2007). The model residuals were analyzed to check the suitability of the error distribution and model fit.

To determine the effects of carcass presence and decomposition on the longevity of larval *T. molitor* (bait insect), we performed survival analyses for the two sets of distance data (vertical and horizontal). Survival times of bait insects (in days) were analyzed using the Weibull distribution. Model simplifications were again performed by contrast analyses. Differences between survival times for the three evaluation periods were evaluated by chi‐squared tests (Crawley, 2007). A final analysis to determine the effects of the “evaluation periods” and “distances from carcass” on the continuous response variable “median survival time” was run using the median survival times obtained from survival analyses. Thus, two models were constructed independently (for depth and horizontal distances) and analyzed by GLM. Model simplification was performed as described above.

## 3 RESULTS

A descriptive record of carcass decomposition and insect activity is provided here, to afford the reader a context in which to interpret the results. During decomposition of the pig carcasses, the first visible signs of decay were a green coloration of the abdomen mixed with bloating, verified between 12 and 20 hr after placement of the carcass on the ground. The general aspects of carcasses in the “During” period (exposed for ca. seven days) were characterized by a liquefied and hence moist mass of tissues with intense insect activity. During this evaluation period (active to advanced decomposition), the soil surrounding the carcass was inundated by moisture and by exudates from the carcass and from insect activity. A great amount of the carcass's biomass was consumed by necrophagous insects and we observed intense soil bioturbation by fly larvae abandoning the carcass to pupate, in addition to nesting activities of beetles. Approaching ten days, the pig carcasses were completely decomposed. After 30 days of exposure, the “After” period, only hair and nail remains persisted above ground, along with the skeletons. Little insect activity was registered in this phase, but there were dozens of Scarabaeinae nests near dry carcass remains.

### 3.1 Influence of decomposition and depth beneath carcass on microorganisms and longevity of bait insects

**Figure 3:**
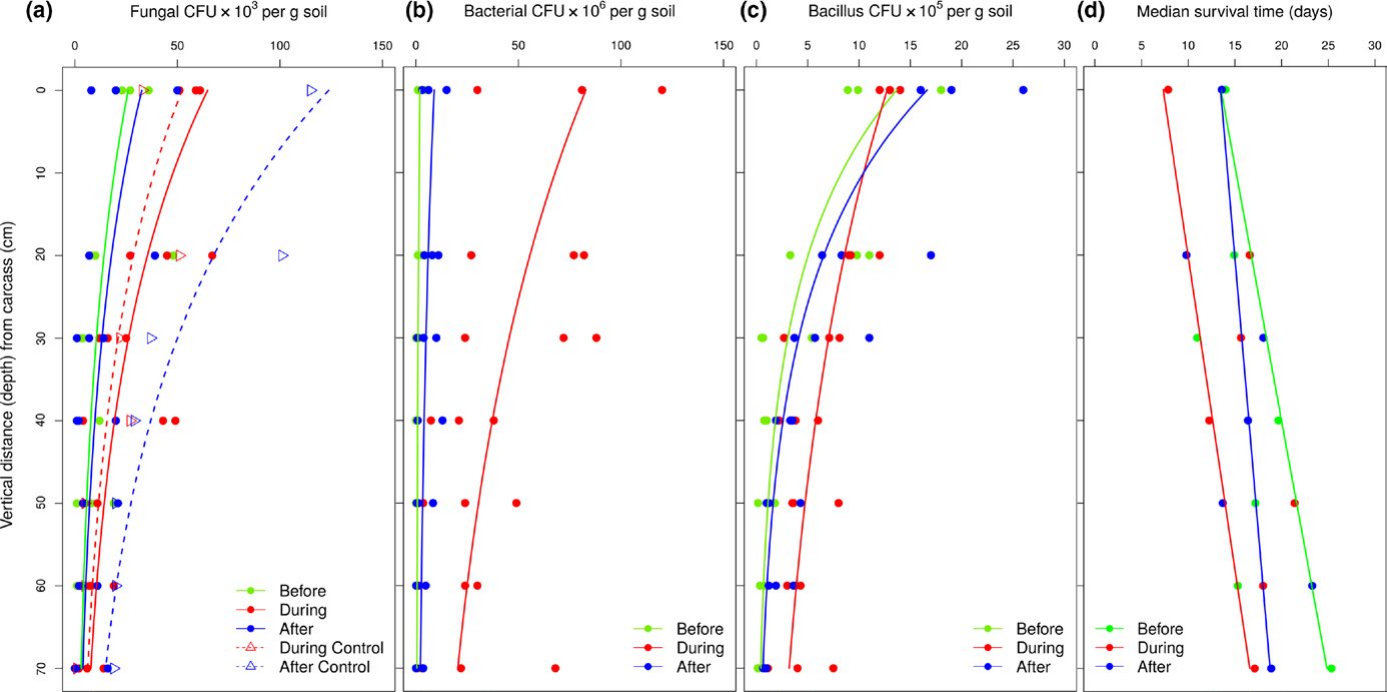
Potential risk of infection imposed on necrophagous insects represented by the effects of carcass decomposition (as measured by the evaluation periods, “Before,” “During,” and “After”) and depth (distances from the carcass) on the density of soil microorganisms (as numbers of colony‐forming units, CFU, per gram of soil): (a) fungal densities (CFUs ×10^3^ per g soil). There was no significant difference in densities of hypocrealean fungi between “Before” and “After” periods (*p* = .392). Despite an increase during the apex of decomposition (“During” period), the hypocrealean density decreased to similar levels without influence of the carcass (i.e., “Before” period) after total decomposition. Controls correspond to sampling in an area without carcass; (b) density of nonfastidious bacteria (CFUs ×10^6^ per g soil); and (c) bacillus densities (CFUs ×10^5^ per g soil). In all microbial groups, the densities decreased with depth, while the survivorship increased (d). In (d), the effect of carcass decomposition and depth on median survival time of the bait insects (*Tenebrio molitor* larvae) placed in contact with soil sampled from around the carcass (according to vertical transect, Figure 2A). Median survival times (days): “Before” = 22.1; “During” = 13.8; and “After” = 18.7 (vide Figure S2a, upper right side). Larval mortality was assessed every two days. Curves represent the evaluation periods according to each graph legend; the total number of CFUs must be multiplied by the dilution factor expressed on the *y*‐axis in (a), (b), and (c). Details of the statistical analyses are presented in the text

Densities of hypocrealean fungi, measured as CFUs per gram of soil from samples, decreased with depth (to 70 cm) for all evaluation periods (Figure 3a). Hypocrealean density was influenced by both depth ($\chi_{\left[1\right]}^{2}$ = 684.85, *p* < .001) and evaluation period ($\chi_{\left[4\right]}^{2}$ = 416.88, *p* < .001), but there was no interaction between these variables ($\chi_{\left[4\right]}^{2}$ = 8.81, *p* = .912). In the second sampling period (“During”), fungal growth coincided with greater rainfall (Appendix S2). There was no significant difference in hypocrealean densities between “Before” and “After” periods ($\chi_{\left[1\right]}^{2}$ = 6.251, *p* = .392), while “During” ($\chi_{\left[1\right]}^{2}$ = 157.25, *p* < .001), “During Control” ($\chi_{\left[1\right]}^{2}$ = 33.162, *p* = .049), and “After Control” ($\chi_{\left[1\right]}^{2}$ = 356.79, *p* < .001) were significantly different from the other evaluation periods “Before” and “After”, with higher hypocrealean densities in all distances evaluated. Hypocrealean densities (mean ×10^3^ ± *SE* CFUs per g soil) were as follows: “Before” = 10.24 ± 2.87, “During” = 25.48 ± 4.78, “During Control” = 20.57 ± 6.89, “After” = 12.86 ± 3.12, and “After Control” = 48.71 ± 15.57.

The densities of nonfastidious cultivable bacteria isolated from soil samples also decreased with depth ($\chi_{\left[1\right]}^{2}$ = 206.59, *p* < .001) (Figure 3b). There was also an effect of evaluation period ($\chi_{\left[1\right]}^{2}$ = 1379.43, *p* < .001), but there was no interaction between these two variables ($\chi_{\left[2\right]}^{2}$ = 2.019, *p* = .882). The negative relation between density and depth was greatest during the apex of decomposition (“active decay”, “During” period), where densities were highest in all evaluated distances (Figure 3b). After this period, bacterial densities decreased a little, but were still higher than in the absence of a carcass (“Before”). Among the depth samples, densities were significantly different between one other in all evaluation periods (“Before” vs. “After”: $\chi_{\left[1\right]}^{2}$ = 52.01, *p* < .01; “During” vs. “After”: $\chi_{\left[1\right]}^{2}$ = 737.64, *p* < .001). Bacterial densities (mean ×10^6^ ± SE CFUs per g soil) for the evaluation periods were as follows: “Before” = 1.02 ± 0.31, “During” = 42.49 ± 7.30, and “After” = 4.60 ± 1.01.

Regarding cultivable bacilli isolated from soil samples, once again, densities decreased with depth in all evaluation periods, with highest densities in the “active decay” (“During”) period (Figure 3c). Bacillus densities were influenced by depth ($\chi_{\left[1\right]}^{2}$ = 202.052, *p* < .001) and evaluation period ($\chi_{\left[2\right]}^{2}$ = 22.041, *p* < .001), and there was also an interaction detected between these variables ($\chi_{\left[2\right]}^{2}$ = 25.125, *p* < .001). Bacillus densities were significantly different from one another in all evaluation periods (“During” vs. “After”: $\chi_{\left[2\right]}^{2}$ = 15.779, *p* < .001; “Before” vs. “After”: $\chi_{\left[2\right]}^{2}$ = 18.062, *p* < .01). Mean densities (mean ×10^5^ ± *SE* CFUs per g soil) were as follows: “Before” = 3.69 ± 1.08, “During” = 6.57 ± 0.84, and “After” = 6.55 ± 1.56. The interaction between distance and evaluation period occurred immediately below the carcass (to 10 cm depth), with “After” having a greater bacillus density than other periods. However, this scenario changed quickly past a depth of 20 cm, with a higher density in the “During” period, thus decreasing in the “After” period to levels near to the “Before” period (i.e., without an influence of the carcass) (Figure 3c).

The median time of survivorship of the mealworm larvae (*T. molitor*) in soil samples from the depth assays increased with the distance from the carcass (i.e., with depth: $\chi_{\left[1\right]}^{2}$ = 159.41, *p* < .001) (Figure 3d). The lowest survivorship was observed during the apex of decomposition (“During”), increasing a little after total decomposition (“After”). However, the longevity of larvae was highest without the carcass (“Before”) (Figures 3d and S2a). Survivorship varied with depth (*p* < .001) and with evaluation period ($\chi_{\left[2\right]}^{2}$ = 188.53, *p* < .001), and there was also an interaction between these variables ($\chi_{\left[2\right]}^{2}$ = 13.41, *p* < .001). Survivorships were significantly different from each another in all evaluation periods (“Before” vs. “After”: $\chi_{\left[2\right]}^{2}$ = 6.103, *p* = .013; “During” vs. “After”: $\chi_{\left[2\right]}^{2}$ = 18.354, *p* < .001) (Figures 3d and S2a). Median survival times were as follows: “Before” = 22.1 days (interquartile range, IQR = 18.9–25.8), “During”  = 13.8 days (IQR = 11.8–16.2), and “After”  = 18.7 days (IQR = 16.0–21.9) (Figure S2a, upper right side).

### 3.2 Influence of decomposition and horizontal distances from carcass on microorganisms and longevity of bait insects

**Figure 4:**
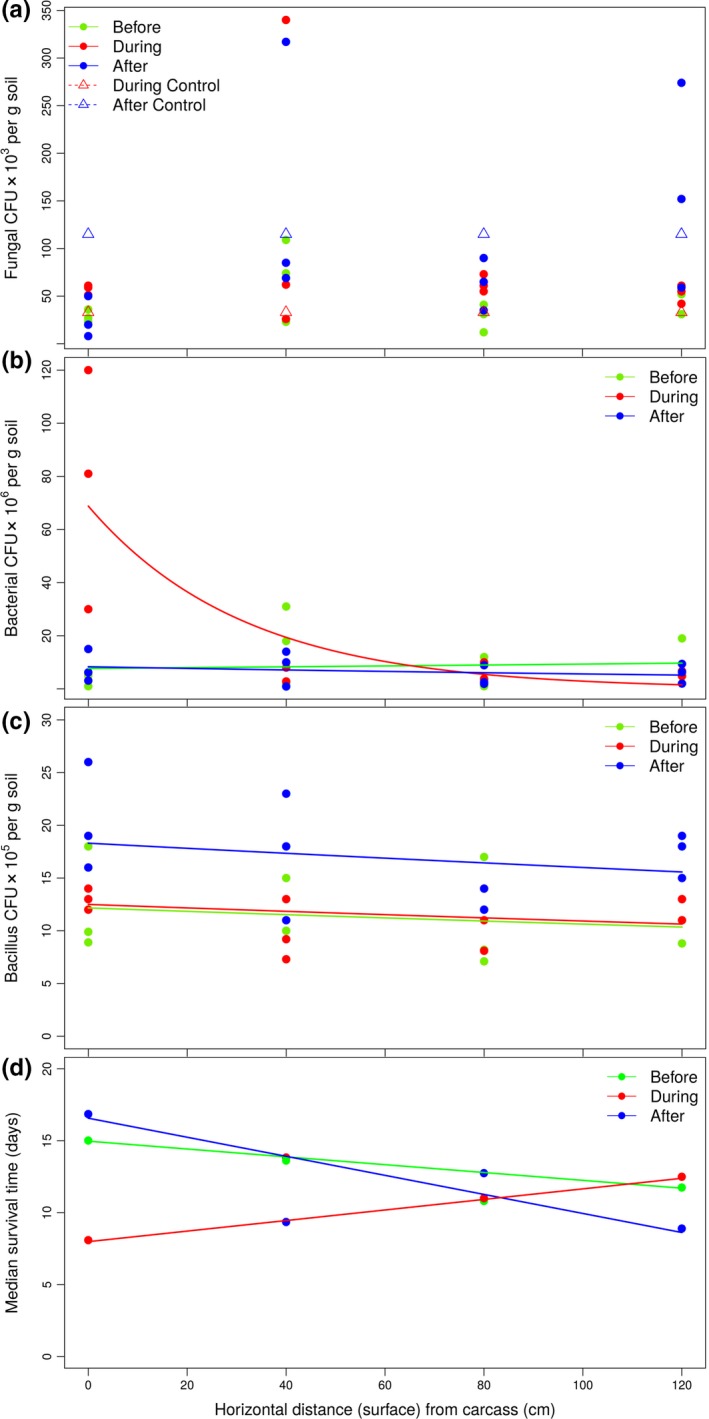
Potential risk of infection imposed on necrophagous insects represented by the effects of carcass decomposition (as measured by the evaluation periods, “Before,” “During,” and “After”) and horizontal (soil surface) distances from the carcass on the density of soil microorganisms (as numbers of colony‐forming units, CFU, per gram of soil): (a) fungal densities (CFUs ×10^3^ per g soil). Neither distance (*p* = .531) nor carcass decomposition (*p* = .054) influenced Hypocreales densities in the superficial layer of soil. Controls correspond to samplings in an area without carcass; (b) densities of nonfastidious bacteria (CFUs ×10^6^ per g soil). There were no significant differences in the bacterial densities between “Before” and “After” periods (*p* = .072). However, during the apex of decomposition, the higher densities of bacteria below the carcass are noteworthy (*p* < .001); (c) bacillus densities (CFUs ×10^5^ per g soil). In this group, the densities were not influenced by distance from the carcass (*p* = .189). Also, there was no difference in the bacterial densities between “Before” and “During” periods (*p* = .823), with the highest bacillus density observed after total decomposition (*p* < .001). In (d), the effect of carcass decomposition and distance from carcass on median survival time of bait insects (*Tenebrio molitor* larvae) placed in permanent contact with soil sampled from around the carcass (according to horizontal transects, Figure 2a,b). There was no significant difference in the larval survivorship between “Before” and “After” periods (*p* = .115), with a median survival time of 14.8 days (vide Figure S2b, upper right side). During the apex of decomposition (“During” period), there was a decrease in larval survivorship, with a median survival time of 11.3 days. Larval mortality was assessed every two days. Curves represent the evaluation periods according to each graph legend; the total number of CFUs must be multiplied by the dilution factor expressed on the *y*‐axis in (a), (b), and (c). Detailed information of the statistical analyses is presented in the text

Densities of putative entomopathogenic fungi (Hypocreales) isolated from soil samples (to 120 cm distance from the carcass) were not influenced by distance ($\chi_{\left[2\right]}^{2}$ = 23.97, *p* < .531) or by evaluation period ($\chi_{\left[4\right]}^{2}$ = 523.01, *p* = .054), and there was no interaction between these variables ($\chi_{\left[4\right]}^{2}$ = 139.36, *p* = .683) (Figure 4a). Comparing hypocrealean densities with distance, the distribution was uniform, thus being similar immediately below (0 cm) or at 120 cm from the carcass, for example. Hypocrealean densities (mean ×10^3^ ± SE CFUs per g soil) were as follows: “Before”  = 40.83 ± 7.71, “During”  = 78.83 ± 23.98, “During Control” = 33.0, “After” = 102.00 ± 28.31, and “After Control” = 115.0.

Densities of nonfastidious cultivable bacteria were influenced by distance from the carcass ($\chi_{\left[1\right]}^{2}$ = 193.81, *p* < .001) and by evaluation period ($\chi_{\left[2\right]}^{2}$ = 150.76, *p* < .001), while there was also an interaction between these variables ($\chi_{\left[2\right]}^{2}$ = 150.35, *p* < .001) (Figure 4b). Bacterial densities showed a different distribution pattern, highest at 0 cm (immediately below the carcass) during the apex of carcass decomposition (“During” period), and decreasing quickly to levels comparable to the “Before/After” period (Figure 4b). The interaction between distance and evaluation period close to 70 cm from the carcass was driven by a weak distance effect in the “Before” and “After” periods and a strong distance effect in the “During” period. Bacteria levels were high near the carcass during the apex of decomposition (“During”) and fell away to the same levels as “Before/After” from 40 cm from the carcass. There was no significant difference in bacterial densities between the periods “Before” and “After” ($\chi_{\left[2\right]}^{2}$ = 6.18, *p* = .072), but “During” densities were different from both ($\chi_{\left[1\right]}^{2}$ = 147.68, *p* < .001). Mean densities of the nonfastidious bacteria (mean ×10^6^ ± SE CFUs per g soil) for the three evaluation periods were as follows: “Before” = 8.67 ± 2.79, “During” = 23.80 ± 10.82, and “After” = 6.68 ± 1.39.

Densities of endospore‐forming bacilli were constant across the distances, but reached highest levels after total decomposition of the carcass (“After” evaluation period) (Figure 4c). Bacillus densities were influenced by evaluation period ($\chi_{\left[2\right]}^{2}$ = 17.776, *p* < .001), but not by distance ($\chi_{\left[1\right]}^{2}$ = 1.725, *p* = .189), and there was no interaction between these variables ($\chi_{\left[2\right]}^{2}$ = 0.576, *p* = .75). While there were no differences in bacillus densities between the “Before” and “During” periods ($\chi_{\left[1\right]}^{2}$ = 0.05, *p* = .823), densities in the “After” period were significantly higher ($\chi_{\left[1\right]}^{2}$ = 17.726, *p* < .001) (Figure 4c). Bacillus densities (mean ×10^5^ ± SE CFUs per g soil) were as follows: “Before” = 11.24 ± 1.01, “During” = 11.55 ± 0.66, and “After” = 16.92 ± 1.31.

Median survival times of bait insects (*T*. *molitor* larvae) in samples of the superficial soil layer were lower during the apex of carcass decomposition (“During” period), increasing with distance from the carcass (Figure 4d). After total decomposition, the larval longevity was higher near the carcass, decreasing with distance and reaching similar levels to that when carcass was absent, that is, “Before” period. The survivorship of larvae was significantly influenced by distance from the carcass ($\chi_{\left[1\right]}^{2}$ = 8.589, *p* = .003), by evaluation period ($\chi_{\left[1\right]}^{2}$ = 20.579, *p* < .001), and by the interactions between evaluation period and distance ($\chi_{\left[1\right]}^{2}$ = 37.097, *p* < .001) (Figure 4d). There was no significant difference in the median times of larval survivorship between the periods “Before” and “After” ($\chi_{\left[2\right]}^{2}$ = 4.333, *p* = .115), but the survival was lower in “During” period ($\chi_{\left[1\right]}^{2}$ = 9.658, *p* = .002) (Figures 4d and S2b). Median survival times were as follows: “Before/After” = 14.8 days (interquartile range, IQR = 13.0–15.9) and “During” = 11.3 days (IQR = 10.2–12.6) (Figure S2b, upper right side).

### 3.3 Fungal isolates

Considering the order Hypocreales, the most abundant genus isolated by the bait insect method was *Metarhizium* (43%) (also abundant in the pour plate method), followed by *Fusarium* (7%), *Beauveria* (2%), and *Isaria* (=*Paecilomyces*) (1%). [Note that percentages correspond to ratios between the numbers of infected *T. molitor* larvae over the total number of larvae used in the insect baiting experiment.]

## 4 DISCUSSION

We evaluated the microbial densities from soil close to carcasses during their decomposition and the microbial activity or ability to infect and kill insects in contact with this soil. This was to shed light on the hypothesis that the elaborate and diverse nesting behaviors of necrophagous beetles could result from the selective pressure exerted by entomopathogenic microorganisms. Our data have shown an increase in the density and activity of fungi and bacteria (opportunistic or facultative pathogens and spoilers) during the period of decomposition with predominance of the necrophagous insects (i.e., “active decay”). Consistent with these results, the survival extrapolated from the bait insect experiments showed a potential risk of infection of the insects that exploit carcasses. The survivorship of the bait insects decreased when in contact with soil from the apex of decomposition (active to advanced decay), indicating the high activity of those microbes present in that period. For the very first time, we are showing the effects of carcass decomposition on putative entomopathogens. Necrophagous insects would appear to be constantly exposed to high densities of microorganisms (potential pathogens and spoilers) while exploring and using carcasses as a resource for feeding and breeding. However, this exposure (and potential risk of harm) may not threaten insects that are able to employ effective strategies against these dangers, such as the Scarabaeinae.

Here, we have used a beetle species that is not adapted to the carcass environment intentionally to offer a better angle on the potential risks associated with exposure to carcasses to a nonadapted insect such as *Tenebrio molitor*, and hence the possible pattern of selection that Scarabaeinae beetles may have experienced. To illustrate this, Bornemissza (1957) investigated the effect of carcass decomposition on soil‐dwelling arthropods (no associated with carcasses), showing a strong reduction in their diversity due to the “unfavorable conditions” generated by decaying carrion. In evolutionary terms, *Tenebrio* has no history of association with the environment of decaying carcasses so has not been selected to survive in such circumstances; this is of relevance to [try to] understand the susceptibility of nonadapted insects and so the selective forces exerted by this environment that may have led to the present diversity of necrophagous insects. It would be interesting to have used Scarabaeinae in the survival experiment (as a form of positive control as they are adapted to exploit rotting organic matter) to compare with the *Tenebrio* data; however, it is practically not viable as (1) it is quite hard to rear them; (2) their development times extend to months or a year; (3) it is also hard to obtain nests under laboratory conditions; and (4) manipulation of examination of nests would need to be performed destructively as larvae stay inside the brood mass/ball, so collecting survival data is virtually impossible. Therefore, indirect evidence provided by *Tenebrio* larvae is a good option to investigate those predictions and potential risks associated with exposure to the carcass environment.

### 4.1 Tunneller beetles may reduce risk from entomopathogens by burrowing deep below the carcass

The general pattern for the assays that considered depth as a variable was a considerable increase in microbial densities in the apex of decomposition (“During” period) in all microbial groups analyzed (i.e., hypocrealean fungi, nonfastidious/opportunistic bacteria, and bacilli). Simultaneously, there was a fast decrease in the survivorship of bait insects, indicating a high potential risk of infection (and of disease). The exudates released by active decay of the carcass provide energy, humidity, and nutrients which alter soil and microbial biomass, activity, and composition (Carter et al., 2007; Cobaugh et al., 2015; Metcalf et al., 2016; Parmenter & MacMahon, 2009). Frequent rainfall during the wet season (Appendix S2) and bioturbation by necrophagous insects carry these nutrients and moisture into the soil profiles, promoting growth of the microbial community even in deeper ground. The gravitational movement of exudates and rainwater (percolation) can also spread microorganisms downwards. Thus, the pulse of nutrients and expansion of microbial community intensifies the activity of many potential pathogens and opportunistic microbes, decreasing the survivorship of soil fauna which do not have defenses against the effects of decomposition (Bornemissza, 1957).

The effects of carcass decomposition below ground become clearer when we compare it to a period without exposure to a carcass. Before placing the pigs in the field, the microbial densities were lower and survivorship was about 1.5 times longer at all distances when compared to the apex of decomposition (above). However, despite this microbial threat, the necrophagous insects are very abundant and diverse in this period of decomposition, suggesting adaptations to exploit this microbe‐rich resource. To find insect cadavers from decaying fauna, such as beetles infected by microbial pathogens, is a quite rare which also indicates resistance or other defensive mechanisms in these insects.

After one month of carcass exposure and total decomposition, the microbial densities along depths return to levels close to the environment without the carcass. In some microbial groups analyzed, the return of densities to initial conditions is swift, such as in the hypocrealean fungal community (Figure 3a), which had no difference between the densities of the “Before” and “After” periods. The same tendency occurs with the nonfastidious bacteria (generally opportunistic ones) and bacilli, especially at greater depths. The impact of decomposition affects the microbial groups in different ways, and probably the causes of changes are mainly related to alterations of soil properties (Metcalf et al., 2016), microbial competition (Janzen, 1977), insect activity (Duarte et al., 2017; Pöppel, Vogel, Wiesner, & Vilcinskas, 2015), and the end of the nutrient influx. After 30 days of exposure, the nutrients released by the carcasses are depleted and the soil below the carcasses is saturated by microbial and insect activities. Reconsidering the analysis of the hypocrealean fungal community, their abundance continued increasing in the area without a carcass, possibly due to continued rainfall (Appendix S2), while after total decomposition of the carcasses, there was a decrease in levels of these fungi. Necrophagous insects are not attracted to the dry remains of carcasses in this period of decomposition. Most scarabaeine have already constructed their nests by then, and tunnellers' nests may be protected by this reduced number of potential pathogens after total decomposition.

Microbial densities increase substantially during the period of peak decomposition, and this is accompanied by a decrease in the survivorship of insects (possibly as a consequence). Nevertheless, this effect is reduced with depth. This is of particular importance for necrophagous burying insects, such as paracoprid scarabaeine beetles (but also for the burying beetles *Nicrophorus* spp. (Silphidae)). They construct nests below the food source (carcass or dung) at different depths according to the species, soil type, and moisture content (Cambefort & Hanski, 1991; Edwards & Aschenborn, 1987; Halffter & Edmonds, 1982). The tunneller's nest contains a chamber that is filled with the amount of food necessary for the development of its offspring. The deeper the nest, the lower the amount of microbes, which decreases the possibility of infection and contamination or spoilage of brood food by opportunistic microorganisms. The deeper profiles of soil present lower and less variable temperatures (Anderson & Coe, 1974; Souza, 2011). Under these conditions, microbial activity is reduced, decreasing the rate of decomposition (Anderson & Coe, 1974) and consequently preserving the nutritional characteristics of the food mass during larval development. Thus, the distance (depth) may be a defensive strategy against the effects of carcass decomposition, such as high probability of infection and also against food spoilage. The relocation of food by tunnellers reduces the contact of food with spoiler microbes (most opportunistic nonfastidious bacteria), preserving the brood food besides avoiding most pathogens of insects in‐depth profiles of soil.

Clay soil is an extra protection used by beetles against microbial invasion. Deeper forest soil layers have high clay contents, and this can be used by beetles as a physical protection to construct smooth tunnels, to mold nest chambers, and to cover the brood mass (Halffter & Edmonds, 1982). The high clay content can stabilize microbial biomass, because bacteria and fungi cannot enter between most clay soil particles (more than 90% according to Oades, 1988). According to Halffter and Matthews (1966), the soil shell may help to avoid desiccation and inhibit fungal growth; while Edmonds (1983) and Davis (1989) pointed out that tunnellers have the strategy of covering the brood mass with a bulky–compact soil shell, as they do not remain to provide care to their brood. This soil shell may work as parental care slowing down fungal invasion (Halffter & Edmonds, 1982). Thus, beetles are protected from disease by little exposure to microbes at depth and by strategies such as wall plastering of the nest chamber and clay soil coat separating the brood balls, which may also serve to limit spread of disease between siblings.

An additional point to be considered is that the nests of tunneller beetles can be indirectly protected by microbial competition and necrophagous insect activity, which releases exudates with antimicrobials, for example, fly larvae (Pöppel et al., 2015), or by‐products with microbial toxicity (Janzen, 1977). They may also be protected indirectly by intense insect visitation of carcasses, which dilutes the *per capita* infection probabilities.

### 4.2 Roller beetles may reduce risk from entomopathogens using another defensive strategy than nesting away from the carcass due their high exposure to pathogens above ground

There was no general pattern of response in the microbial groups analyzed for horizontal distances from carcass (on topsoil). Each microbial group presented a different response to the presence of a carcass and its decomposition. The changes caused by decomposition on the microbial community seem to be quickly diluted or overlaid on topsoil, except in the endospore‐forming bacteria (discussed below). After total decomposition (i.e., 30 days after carcass exposition), the effect of carcass decay on the opportunistic nonfastidious bacteria and on bait survivorship was similar to the period before exposure to the carcass (and different from the apex of decomposition). Some factors can be attributed to this, such as the common rainfalls of the wet season, draining the nutrients released by the carcass and the microbial CFUs; the intense visitation and bioturbation promoted by necrophagous insects, spreading the microbes; and by succession of the microbial community, which is expected to be fast on topsoil after corpse rupture (Metcalf et al., 2016).

Horizontal distance from carcasses had no influence per se on the distribution and abundance of putative pathogens of insects, that is, hypocrealean fungi and bacilli. This means that in these groups, even below the carcass, the density is similar to that at 1.2 m from the carcass (i.e., the furthest distance evaluated). Besides this, the amount of hypocrealean fungi did not change with the presence of the carcass, and the bacillus density was remarkably high only after total decomposition, maintaining its homogeneous distribution among distances. After total decomposition, the environment surrounding dry remains of a carcass becomes toxic for many organisms (Bornemissza, 1957), including microbes (Janzen, 1977). However, bacteria which produce endospores such as *Bacillus* spp. (class Bacilli) are extremely resistant to extreme environmental conditions (Nicholson, Munakata, Horneck, Melosh, & Setlow, 2000), staying for long periods on topsoil after growth when conditions were favorable. Nevertheless, the abundance of putative entomopathogenic bacilli observed after total decomposition may not affect the necrophagous beetles, because they frequent carcasses predominantly in the initial stages of decomposition. However, the structure of the community can change qualitatively during the apex of decomposition and favor more virulent fungal and bacterial species without significant changes in the densities between the periods of evaluation. Pechal et al. (2013) recorded the phylum Firmicutes, which includes bacilli, as the second most abundant related to decaying pig carcasses (also using *Sus scrofa* 14–18 kg). They saw that abundance of Firmicutes increased as decomposition progressed. This general pattern is consistent with our results for endospore‐forming bacilli, highlighting the potential risks of infection in this scenario.

The release of nutrients and moisture during the apex of decomposition showed significant impacts on the opportunistic nonfastidious bacteria, mainly below the carcass (at 0 cm). However, the abundance of these bacteria correlates negatively with distance. Probably due to its opportunistic nature, this group of bacteria was more sensitive to pulses of nutrients and less affected by microbial competition and variations in soil biochemistry. The decomposition ending and consequent ending of the pulse of nutrients also affect these bacteria rapidly, showing similar bacterial density in the period without the carcass. Moreover, antimicrobials and toxic exudates from necrophagous fauna (Pöppel et al., 2015) and other microbes during decomposition of the carcass must also contribute to the reduction in abundance in this bacterial community. This group of bacteria showed similar responses among depths, but do not represent a direct threat to the necrophagous insects. Most of these bacteria are not entomopathogenic, but can spoil or contaminate with toxins the meat that larvae of scarabaeine beetles will eat during their development. Because of this, the presence and response of these bacteria during decomposition are also important to be considered. The relocation of food by rollers may also be a protective strategy, which reduces the contact of food with the highest amount of spoiler microbes, preserving the brood food from them. However, rollers need to use additional defensive strategies (discussed below) due to the natural distribution of putative pathogens of insects predominantly on topsoil, also seen in the lower survivorship of bait insects on topsoil samples than in samples from deep soil layers.

The significant reduction in the survivorship of bait insects during the apex of decomposition on topsoil, when necrophagous insects are most abundant, reveals the dangerous environment surrounding a carcass, in terms of the probability of infection on the soil surface. Bait survivorship also increased with distance in this period of carcass decay, possibly indicating an increase in the pathogens' activity. Following this reasoning, the impact on survivorship may be attributed to increases in more virulent species of Hypocreales and/or bacilli, for example, and additional experiments should clarify the threat of disease that necrophagous insects are submitted to in the forest. Above ground (superficial soil layer), the possibilities of infection do not decrease with distance from the carcass, which may explain the existence of other defensive strategies during the nidification of the roller beetles (Halffter & Edmonds, 1982; Halffter & Matthews, 1966), such as antimicrobial secretions (Cortez‐Gallardo & Favila, 2007), a brood ball covered with a soil layer (Cartwright, 1949; Howden & Ritcher, 1952), and constant parental care during larval development (Edwards, 1988; Favila, 1993; Halffter, 1977).

### 4.3 Strategies against the potential risk of infection

Overall, the survivorship of bait insects was greater in deep soil than on the surface, highlighting the risky lifestyle of roller beetles in the forest. Forests have naturally higher abundances of insect pathogens than other habitats (Lodge, Hawksworth, & Ritchie, 1996), which may explain the low abundance of roller beetles in tropical forests (Halffter et al., 1992). The rollers' reproductive repertoire includes mainly taking a part of the resource, making a food ball, and rolling it for long distances until placing it in a cup‐shaped nest over soil or buried superficially (Halffter & Edmonds, 1982). During rolling, the beetle's abdomen remains in constant contact with the food ball, which itself is in contact with topsoil that is full of pathogens and opportunistic microbes (see above). The rolling behavior may add many microbial cells to the ball (Figure 1b), including pathogens, and the frequent contact between ball and abdomen could increase the exposure of parents to pathogens. The superficial nests of rollers are also constantly in contact with high amounts of putative pathogens from topsoil, independent of the distance from the carcass.

However, the roller beetles have additional defensive strategies. Each brood ball is covered by a thin soil coat, while the egg placed over the food mass is also enclosed with a soil coat to avoid direct contact between the egg and decaying food (Edmonds, 1983; Halffter & Edmonds, 1982; Halffter & Matthews, 1966). Additionally, parents usually work together and have exocrine glands on their legs and abdomen (sternal and pygidial) that produce protective secretions, which may contain antimicrobials, deterrent substances, or just a form of cement to amalgamate the soil used to cover the food ball (Cortez et al., 2015; Favila, 1993; Pluot‐Sigwalt, 1988b, 1991). The characteristic upside‐down position during rolling (Figure 1b) permits impregnation of secretions in the food ball (Bellés & Favila, 1983; Cortez‐Gallardo & Favila, 2007; Favila, 1988; Pluot‐Sigwalt, 1991), but the nature of these secretions remains poorly studied. Recently, a great diversity of compounds was identified (Cortez et al., 2015) in the secretion of the pygidial gland of the necrophagous roller beetle *Canthon cyanellus cyanellus* (Pluot‐Sigwalt, 1988a,b, 1991). Some of the identified compounds have potentially defensive, preservative (retarding spoilage), and antimicrobial functions (Cortez et al., 2015; Cortez‐Gallardo & Favila, 2007). This secretion combined with the soil coat and parental care seems to be an effective arsenal which allows the insects to overcome the threats of infection in topsoil environments.

Contamination of larval food by spoiler microbes also can be restricted by extensive parental care. The roller species mostly stay taking care of the brood ball, removing fungal hyphae and other opportunistic microorganisms, and repairing the soil coat. Favila (1993) reported that brood balls of *Canthon cyanellus cyanellus* were covered by fungi when females were absent from the nest. Other studies also highlighted the extreme importance of parental care among scarabaeine beetles, pointing out the relevance of care in avoidance of fungal attack (Cortez‐Gallardo & Favila, 2007; Edwards, 1988; Halffter et al., 1996; Huerta & Halffter, 2000; Kryger, Cole, Tukker, & Scholtz, 2006). However, it should be noted that there is no record of significant bacterial invasion or damage in brood balls, even when the parents are removed experimentally after oviposition. All reports are related to opportunistic fungal invasions, which first attack the larval food, without any record of adult infection. Thus, the rolling strategy combined with soil coat, defensive secretions, and parental protection appears to be a good way to limit microbial threats (pathogens and food spoilers).

Considering the lifestyle of tunneller beetles and the higher mortality of insects in topsoil than in deeper soil, independently of carcass presence and decomposition, it is possible to consider that tunnellers present the best nesting strategy against harmful microbes of the forest. Probably, this can explain their success in terms of higher diversity and dominance in forests (Halffter et al., 1992) in comparison with rollers or dwellers. The latter group must be the most disease‐threatened beetle as they do not relocate food to eat and breed, and stay intimately involved inside or between the resource (carcass or dung) and soil surface (in the soil connected with the food resource). Consequently, dwellers should need potent mechanisms of defense to exploit and reproduce in the carcass, avoiding disease especially during the pupal stage and emergence. The nesting strategy adopted by most dwellers is to lay many eggs in a chamber constructed in the resource, covering the eggs with a soil coat and providing parental care (Davis, 1989; Halffter & Edmonds, 1982; Kingston & Coe, 1977). Dwellers are also weak competitors that arrive late to the carcass and choose old resources already used by fly larvae (Doube, 1990), or with low densities of competitors (Doube, 1991; Halffter & Edmonds, 1982). Thus, all these characteristics could explain the lower diversity of dwellers than tunnellers and rollers in tropical forests (Halffter et al., 1992).

This study demonstrates that the microbial community of putative entomopathogens and opportunistic microbes, under effects of carrion decomposition (i.e., availability of moisture and nutrients), may become more numerous and active (perhaps even more virulent), probably exerting selective pressure on necrophagous insects. In both distance assays (vertical and horizontal), survivorship was less during active decay. Thus, we suggest that the possibility of infection may drive different breeding strategies as forms of behavioral immune defense. Historically, the hypothesis more accepted for the different nesting behaviors observed in Scarabaeinae is that the competition among beetles favors the relocation of food. However, based on our data, we propose that the effect of the resource decomposition in microbial communities is also an important selective pressure on the life history of Scarabaeinae. This selective force may drive the diversity in nesting strategies with elaborate forms of care among scarabaeine beetles to overcome the threat from potential pathogens and spoilers present in the resource used to feed and reproduce. So their nesting behaviors seem very effective against pathogens and opportunistic microbes, reflected in the low incidence of disease or fungal infestation in brood ball/mass and nest chambers in natural conditions. Despite our focus on carrion feeders, we expect that the same occurs with coprophagous beetles (dung feeders), because both resources have similar characteristics (i.e., rich in nutrients, ephemeral, unpredictable, and disputed with other organisms).

## CONFLICT OF INTEREST

None declared.

## AUTHOR CONTRIBUTIONS

V.S.F. and S.L.E. conceived and designed the research. V.S.F. and V.B.R. performed the experiments. V.S.F., V.B.R., and S.L.E. analyzed the data. V.S.F. wrote the original draft. V.S.F. and S.L.E. reviewed and edited the manuscript. All authors approved the final version of the manuscript.
