# Supplementary material for: Nesting strategies and disease risk in necrophagous beetles
Source: Ecol Evol. 2018 Feb 19;8(6):3296–310. doi: 10.1002/ece3.3919 (PMC5869311; doi:10.1002/ece3.3919)
Supplement: Supplementary file 1 [file ECE3-8-3296-s001.docx]

**APPENDIX S1 – Experimental Area**

**Figure S1. Sampling location.** Site where the field experiment was conducted, *Estação de Pesquisa, Treinamento e Educação Ambiental Mata do Paraíso*, run by the Universidade Federal de Viçosa, Viçosa city, State of Minas Gerais, Southeast Brazil. This research station comprises ca. 200 ha of Atlantic Forest biome: **(a)** Google map of the research station delimited by yellow line, and the red point corresponds to area less irregular topographically at 20° 48’ 18” S, 42° 51’ 20” W, link <https://goo.gl/maps/KYSVCJ3r5Lw>; **(b)** photograph of the forest at the red point marked in (a); and **(c)** lake which belongs to the station, note the forest in the background.

**APPENDIX S2 – Meteorological Data**

**Table S1. Meteorological data on the sampling days.** Data available from the Instituto Nacional de Meteorologia (INMET), Brazil. Station: Viçosa/Minas Gerais (OMM: 83642); latitude: -20.76°; longitude: -42.86°. Accessed on: April/2016 <http://www.inmet.gov.br/portal/>

| **Date**  **(m/d/y)** | **Precipitation**  **(mm)** | **Maximum temperature (°C)** | **Minimum temperature (°C)** | **Average relative humidity (%)** |
| --- | --- | --- | --- | --- |
| ***Dry season ****  04/27/2015 (b)  05/05/2015 (d)  06/01/2015 (a) | 0.0  0.0  0.0 | 24.6  22.4  25.0 | 15.0  18.4  14.5 | 80.0  92.8  84.8 |
| ***Wet season***  01/27/2016 (b) | 8.6 | 31.8 | 21.0 | 77.0 |
| 02/04/2016 (d) | 15.4 | 31.8 | 19.1 | 78.0 |
| 02/29/2016 (a) | 8.1 | 32.4 | 20.6 | 79.5 |

** pilot experiment*

*b, d, a* - periods of evaluation:

*(b) before placing the carcass (0 days after decomposition began)*

*(d) during decomposition (active decay) (5 – 8 days after decomposition began)*

*(a) after total decomposition (skeletonization) (30 days after decomposition began)*

**APPENDIX S3 – Pilot Experiment**

The pilot experiment conducted in the dry season (Appendix 2) was set up with four horizontal transects in each evaluation period (sampling), and was aimed only to access density by the isolating of the Hypocrealean fungi CFUs for logistical reasons. After data analyses, we reduced the number of horizontal transects during periods of evaluation to be performed in the wet season as no significant differences were found between them. This economy of effort allowed us to measure in the main experiment the effects of decomposition in additional microorganisms, such as with endospore-forming Bacilli and cultivable non-fastidious opportunistic bacteria.

The general data of the pilot experiment conducted in the dry season pilot were compared briefly with data from the wet season experiment. The density of putative entomopathogenic fungi (Hypocreales) isolated from the soil samples from depths in the wet season had two times higher density than in the dry season (mean ± SE CFUs): dry = 6.71 ± 2.87 and wet = 16.19 ± 4.23. The same was observed in density of soil samples of the horizontal distances (mean ± SE CFUs): dry = 28.42 ± 4.54 and wet = 73.89 ± 8.82. Additionally, we saw that there was a significant difference in the bait insect survival between seasons (χ²_[1]_ = 37.519, p < 0.001). In general, bait insects lived longer in soil samples from the dry season 19.79 ± 2.89 days (mean ± SE) compared to the wet season 14.37 ± 2.78 days. These results are of relevance because many Scarabaeinae beetles stay below ground during a great part (or all) of the dry season ([e.g. the tunneller Heliocopris dilloni; Kingston & Coe 1977](#_ENREF_4_1)). After the first rains of the wet season, they emerge from the soil to reproduce ([Kingston & Coe 1977](#_ENREF_4_1)) and then construct a nest on the ground, keeping close contact with soil again. The humidity of the wet season can enhance the activity and virulence of soil microbes, making it more advantageous to the insects to stay underground in the dry season (i.e. with low probabilities of infection).

**REFERENCE**

Kingston, T.J. & Coe, M. (1977). The biology of a giant dung-beetle (*Heliocopris dilloni*) (Coleoptera: Scarabaeidae). *Journal of Zoology*, 181, 243-263.

**APPENDIX S4 – Longevity of Insects**

**Figure S2. Survival probability of the bait insects.** To confirm the presence or killing power of putative entomopathogens in the soil samples which constitute a threat to necrophagous insects, we evaluated the longevity of bait insects (*Tenebrio molitor* larvae) placed in contact with soil sampled from around the carcass (according to vertical and horizontal transects, Figure 2): **(a)** the survivorship of mealworm larvae in soil samples from the depth assays was lowest during the apex of decomposition (“During”), increasing a little after total decomposition (“After”). However, the longevity of larvae was highest without the carcass. Longevity increased with depth (χ²_[1]_ = 36.86, p < 0.001), and varied with evaluation period (χ²_[2]_ = 33.362, p < 0.001), but there was no interaction between these variables (χ²_[2]_ = 5.33, p = 0.07). Survivorships were significantly different from one another in all evaluation periods (“Before” *vs.* “After”: χ²_[1]_ = 6.103, p = 0.013; “During” *vs.* “After”: χ²_[1]_ = 18.354, p < 0.001). Median survival times, in the upper right side were: “Before” = 22.1 days (interquartile range, IQR = 18.9‒25.8), “During” = 13.8 days (IQR = 11.8‒16.2), “After” = 18.7 days (IQR = 16.0‒21.9). **(b)** survival of mealworm larvae in samples of the superficial soil layer was also lower during the apex of carcass decomposition, while after total decomposition the longevity was similar to that when carcass was absent, i.e. “Before” period. Longevity of larvae was significantly influenced by evaluation period (χ²_[2]_ = 9.859, p = 0.007) and by the interaction between evaluation period and distance (χ²_[2]_ = 25.119, p < 0.001; analysing only distance, this variable did not influenced the survivorship: χ²_[1]_ = 1.524, p = 0.217). There was no significant difference in larval survival times between the periods “Before” and “After” periods (χ²_[2]_ = 4.333, p = 0.115), but the survival was significantly lower during the apex of decomposition (χ²_[1]_ = 9.658, p = 0.002). Median survival times, in the upper right side were: “Before/After” = 14.8 days (interquartile range, IQR = 13.0‒15.9), “During” = 11.3 days (IQR = 10.2‒12.6). Curves represent the evaluation periods. Larval mortality was assessed every two days.
