# Supplementary figures and images for: Nesting strategies and disease risk in necrophagous beetles
Source: Ecol Evol. 2018 Feb 19;8(6):3296–310. doi: 10.1002/ece3.3919 (PMC5869311; doi:10.1002/ece3.3919)

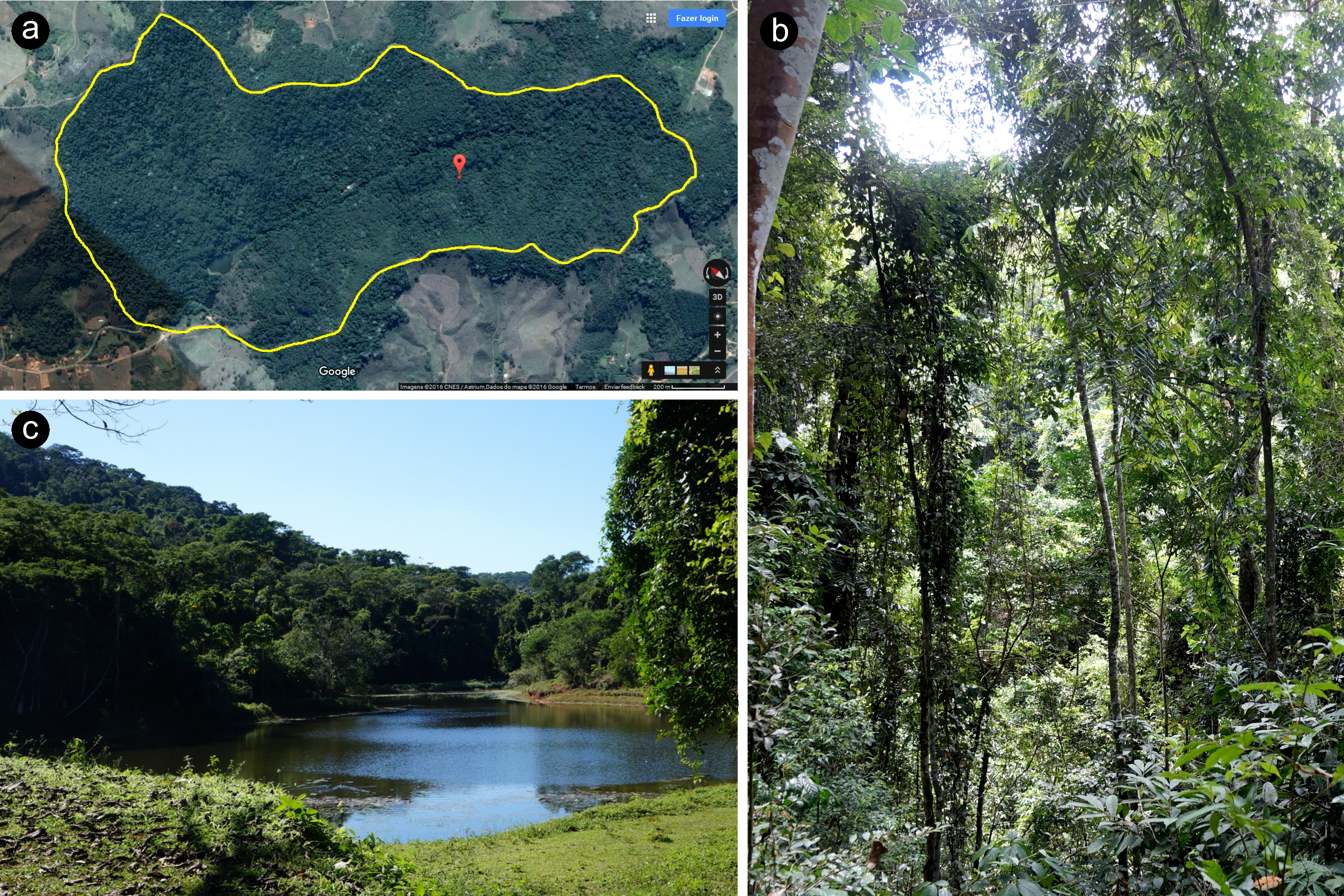

Supplement: Supplementary file 2 [file ECE3-8-3296-s002.png]

**(a)**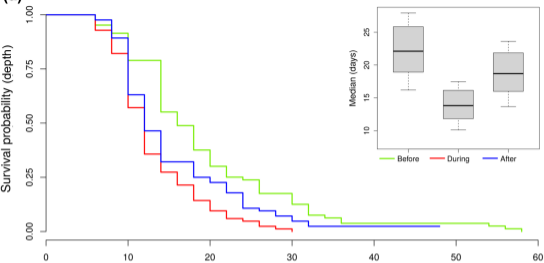**(b)**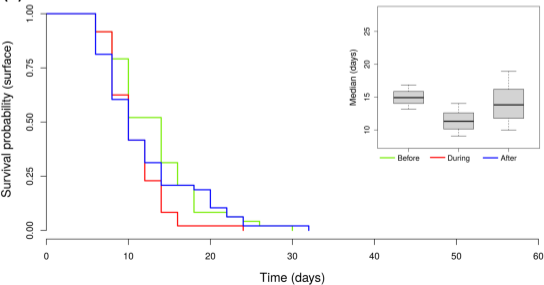

Supplement: Supplementary file 3 [file ECE3-8-3296-s003.pdf]
